# Auditory Stream Segregation and Selective Attention for Cochlear Implant Listeners: Evidence From Behavioral Measures and Event-Related Potentials

**DOI:** 10.3389/fnins.2018.00581

**Published:** 2018-08-21

**Authors:** Andreu Paredes-Gallardo, Hamish Innes-Brown, Sara M. K. Madsen, Torsten Dau, Jeremy Marozeau

**Affiliations:** ^1^Hearing Systems Group, Department of Electrical Engineering, Technical University of Denmark, Kongens Lyngby, Denmark; ^2^Department of Medical Bionics, The University of Melbourne, Melbourne, VIC, Australia; ^3^Bionics Institute, East Melbourne, VIC, Australia

**Keywords:** auditory scene analysis, segregation, cochlear implant, auditory attention, event-related potentials, build-up

## Abstract

The role of the spatial separation between the stimulating electrodes (electrode separation) in sequential stream segregation was explored in cochlear implant (CI) listeners using a deviant detection task. Twelve CI listeners were instructed to attend to a series of target sounds in the presence of interleaved distractor sounds. A deviant was randomly introduced in the target stream either at the beginning, middle or end of each trial. The listeners were asked to detect sequences that contained a deviant and to report its location within the trial. The perceptual segregation of the streams should, therefore, improve deviant detection performance. The electrode range for the distractor sounds was varied, resulting in different amounts of overlap between the target and the distractor streams. For the largest electrode separation condition, event-related potentials (ERPs) were recorded under active and passive listening conditions. The listeners were asked to perform the behavioral task for the active listening condition and encouraged to watch a muted movie for the passive listening condition. Deviant detection performance improved with increasing electrode separation between the streams, suggesting that larger electrode differences facilitate the segregation of the streams. Deviant detection performance was best for deviants happening late in the sequence, indicating that a segregated percept builds up over time. The analysis of the ERP waveforms revealed that auditory selective attention modulates the ERP responses in CI listeners. Specifically, the responses to the target stream were, overall, larger in the active relative to the passive listening condition. Conversely, the ERP responses to the distractor stream were not affected by selective attention. However, no significant correlation was observed between the behavioral performance and the amount of attentional modulation. Overall, the findings from the present study suggest that CI listeners can use electrode separation to perceptually group sequential sounds. Moreover, selective attention can be deployed on the resulting auditory objects, as reflected by the attentional modulation of the ERPs at the group level.

## Introduction

Many daily listening scenarios involve multiple sound sources. Thus, to selectively listen to a single person’s voice among many, or to a melody in a complex musical arrangement, the listener needs to parse the sounds in the complex auditory scene and group them into meaningful auditory objects or streams (e.g., McDermott, 2009). This process is known as auditory scene analysis (Bregman, 1990). Hearing impairment may affect the process of object formation and thus, hearing-impaired (HI) listeners generally perform worse than normal-hearing (NH) listeners in complex listening scenarios (e.g., Mackersie et al., 2001; Oxenham, 2008). This is the case even when hearing aids or cochlear implants (CIs) are used to make the signals audible (e.g., Nelson et al., 2003). Most current CIs convey spectral information through *place cues*, whereby different frequency bands of the acoustic signal are used to stimulate different electrodes at a given pulse rate. It is unclear to what extent CI listeners can use place cues in the process of object formation. Thus, a better understanding of the role of place cues in the process of object formation would be beneficial to overcome the challenges that CI listeners experience in complex listening scenarios.

Two main processes have been described in auditory scene analysis: auditory stream integration (the perceptual grouping of multiple sound events into a single stream) and auditory stream segregation (the perceptual grouping of multiple sound events into several streams). In NH listeners, these processes have been studied in experiments that employ sequences of repeating, sequentially-presented sounds. These sounds differ in various acoustic properties, such as frequency content (e.g., Bregman and Campbell, 1971; Van Noorden, 1975) or temporal envelope (e.g., Iverson, 1995; Cusack and Roberts, 2000). Large differences between the sounds facilitate segregation, whereas small differences promote integration. Furthermore, it has been suggested that sequential stream segregation may be directly related to the degree of the perceptual difference between the sounds (Moore and Gockel, 2002, 2012).

In addition to stimulus-driven effects, stream segregation can be influenced by cognitive factors, such as attention. Van Noorden (1975) investigated the perceptual organization of sounds using sequences of alternating low- and high-frequency pure tones. The segregation threshold varied with the intention of the listener: a smaller frequency difference was needed when the listeners were encouraged to segregate the sounds (i.e., *voluntary* stream segregation) than when the listeners were encouraged to integrate them (i.e., *obligatory* stream segregation). From these results, Van Noorden defined the *fission boundary* as the frequency separation below which the sounds could no longer be segregated and the *temporal coherence boundary* as the frequency separation above which the sounds could no longer be integrated. Thus, the temporal coherence boundary and the fission boundary represent the thresholds of obligatory and voluntary stream segregation, respectively. The probability of perceiving a segregated percept has been reported to increase over time. This is often referred to as the *build-up effect* of stream segregation (e.g., Van Noorden, 1975; Bregman, 1978; Anstis and Saida, 1985). The build-up effect has been reported to occur both under integration-promoting listening instructions (e.g., Roberts et al., 2008; Thompson et al., 2011) and under segregation-promoting listening instructions (Micheyl et al., 2005; Nie and Nelson, 2015).

Several studies have investigated obligatory stream segregation in CI listeners (e.g., Cooper and Roberts, 2009; Duran et al., 2012; Tejani et al., 2017). These studies varied either the spatial separation between the stimulating electrodes (i.e., place cues) or the pulse rate of stimulation at a fixed electrode (i.e., temporal cues). The manipulation of these parameters is known to elicit perceptual differences in CI listeners (e.g., Eddington et al., 1978; Landsberger et al., 2016). Moreover, it has been suggested that stream segregation may be related to the degree of the perceptual difference between the streams, regardless of whether such perceptual difference was elicited by varying the place or the rate of stimulation (Paredes-Gallardo et al., 2018b). The results from Tejani et al. (2017) were consistent with those from studies in NH listeners, i.e., the probability of experiencing a two-stream percept increased by increasing the electrode separation. Similarly, the results from Duran et al. (2012) suggest that CI listeners experience obligatory stream segregation when only temporal cues are provided. However, Cooper and Roberts (2009) did not observe a build-up effect, suggesting that not all elements of obligatory stream segregation may be experienced by CI listeners.

Cooper and Roberts (2009) also assessed voluntary stream segregation abilities of CI listeners using a melody discrimination task. The listeners were asked to identify a pattern of sequentially activated electrodes (melody) in the presence of interleaved, random distractor sounds. Their results showed that CI listeners were not able to segregate the melody from the distractor sounds, regardless of the electrode separation between the streams. Conversely, other studies suggest that CI listeners can use either electrode separation, pulse rate differences or amplitude modulation (AM) rate differences to voluntarily segregate sequences composed of two repeating and alternating sounds (Hong and Turner, 2009; Paredes-Gallardo et al., 2018a,b). Moreover, a build-up effect has been reported to occur during voluntary stream segregation when using either place or temporal cues (Paredes-Gallardo et al., 2018a,b).

There are several differences between the study of Cooper and Roberts (2009) and those of Hong and Turner (2009) and Paredes-Gallardo et al. (2018a,b). The sequences of sounds were shorter in the study by Cooper and Roberts (2009) than those used in the studies of Hong and Turner (2009) and Paredes-Gallardo et al. (2018a,b). Thus, it is possible that the poor performance reported in the study of Cooper and Roberts (2009) reflects the long time that CI listeners need to build up a segregated percept, even when the attention of the listener is directed toward segregation (Paredes-Gallardo et al., 2018a). Another difference is that Cooper and Roberts used streams composed of different sounds, resulting in a more complex task than those employed in the studies of Hong and Turner (2009) and Paredes-Gallardo et al. (2018a,b) Finally, the stimuli used by Hong and Turner (2009) and Paredes-Gallardo et al. (2018a,b) might have facilitated segregation due to the inclusion of rhythmic cues (for a review, see Bendixen, 2014). Thus, it is unclear whether the poor performance reported by Cooper and Roberts reflects that CI listeners need longer time to build up a two-stream percept or that CI listeners are not able to segregate streams composed of different sounds in the absence of rhythmic cues.

It has been suggested that selective attention operates as a form of sensory gain control, modulating the neural representations of signals in the auditory cortex. Specifically, selective attention has been shown to enhance the event-related potentials (ERPs) evoked by attended sounds and to suppress those evoked by ignored sounds (e.g., Hillyard et al., 1973, 1998; Picton and Hillyard, 1974). Thus, several studies have used recordings of ERPs to investigate the process of object selection and selective attention, tightly related to stream segregation (e.g., Alain and Arnott, 2000; Choi et al., 2013). Moreover, the amount of attentional modulation of the ERPs has been shown to correlate with the listener’s ability to perform an auditory selective-attention task (Choi et al., 2014; Dai and Shinn-Cunningham, 2016; Dai et al., 2018), suggesting a strong link to perception. However, to the authors’ knowledge, no previous study has investigated whether selective attention modulates the ERPs in CI listeners and whether such attentional modulation would correlate with performance in an auditory selective-attention task. If this would be the case, the attentional modulation of the ERPs could be used as an objective tool to assess stream segregation and selective attention in CI listeners.

The present study investigated, in CI listeners, (1) whether electrode separation is a cue for the segregation of streams composed of different sounds, (2) whether a two-stream percept builds-up over time, (3) whether selective auditory attention modulates the amplitude of the ERPs, and (4) whether such attentional modulation of the ERP reflects individual stream segregation abilities and therefore, whether the attentional modulation of the ERPs can be used as an objective tool to assess voluntary stream segregation. Behavioral detection performance was measured in a paradigm where the listeners were required to attend to a series of sounds in the presence of interleaved distractor sounds. A deviant was randomly introduced in the target stream either at the beginning, middle or end of each trial. The listeners were asked to detect sequences that contained a deviant and to report its location within the trial. As in the task described by Cooper and Roberts (2009), the perceptual segregation of the streams should improve performance in the deviant detection task. It was hypothesized that if CI listeners can use electrode separation as a cue to segregate the streams, performance in the deviant detection task would improve with increasing electrode separation between the target and the distractor streams. If CI listeners need time to build up a segregated percept, detection performance should be highest for deviants presented late in the trial. Furthermore, ERPs to the same stimuli were recorded while the listeners performed the behavioral task (active listening) and while they watched a muted movie (passive listening). It was hypothesized that if CI listeners can segregate the streams, then the ERPs evoked by the target stream should be enhanced in the active listening condition compared to the passive listening condition. Conversely, ERPs evoked by the distractor stream should be suppressed in the active listening condition with respect to the passive listening condition.

## Materials and Methods

The experiments took place in a sound-attenuating and electrically shielded booth at the Bionics Institute of Australia and at the Technical University of Denmark. The experiments were conducted in three sessions, each lasting 2 h including short breaks. The first session comprised categorical loudness scaling and loudness matching of the different stimuli, a pitch ranking task, a test run of the detection task in the absence of the distractor stream and a 15–20 min training on the segregation task. The behavioral experiment and the recording of the ERPs took place in the second and third sessions, respectively.

### Listeners

Twelve CI listeners participated in this study. The listeners were aged between 20 and 82 years (mean: 61.3 years, *SD*: 22.2 years; see **Table 1**) and had no residual hearing in the implanted ear. For the bimodal listeners, the contralateral ear was unaided and blocked with an earplug during the experiments. All listeners performed the behavioral task. Listener CI-10 did not participate in the ERP recording session. All listeners provided written informed consent prior to the study and all experiments were approved by the Human Research Ethics Committee of the Royal Victorian Eye & Ear Hospital (reference 14.1180H) and the Science-Ethics Committee for the Capital Region of Denmark (reference H-16036391). All listeners were users of the Cochlear Ltd. (Sydney, Australia) implant. The electrode array of the Cochlear Ltd. implant consists of 22 electrodes where electrode 1 is the most basal electrode and electrode 22 is the most apical one.

**Table 1:** Information about the participants regarding age, gender, onset of deafness, implanted ear, number of years of experience and modality of rehabilitation.

Listener	Age range	Onset of deafness	Implant model (ear)	Years of experience	Modality
CI-1	30–49	Postlingual	CI24RE (right)	4	Bilateral
CI-2	>70	Postlingual	CI522 (left)	1	Bimodal
CI-3	<30	Postlingual	CI522 (left)	1	Unilateral
CI-4	50–69	Postlingual	CI24RE (left)	4	Bimodal
CI-5	>70	Postlingual	CI512 (left)	1	Bimodal
CI-6	>70	Postlingual	CI512 (right)	2	Bilateral
CI-7	50–69	Postlingual	CI24RE (left)	3	Bilateral
CI-8	>70	Postlingual	CI24RE (right)	8	Bimodal
CI-9	>70	Perilingual	CI24RE (right)	3	Bilateral
CI-10	<30	Perilingual	CI24RE (left)	7	Unilateral
CI-11	50–69	Perilingual	CI24RE (left)	8	Bilateral
CI-12	>70	Postlingual	CI24R (left)	14	Bilateral


### Task Description

**FIGURE 1:**
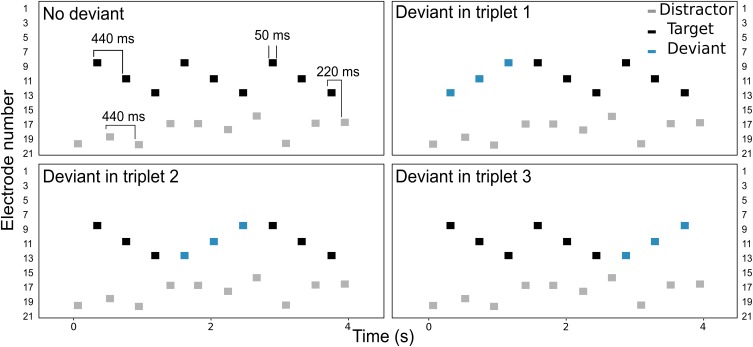
Schematic representation of the electrodogram for each of the four deviant conditions. Black and gray markers represent the target and the distractor sounds, respectively. The deviant triplet is shown with blue markers.

**FIGURE 2:**
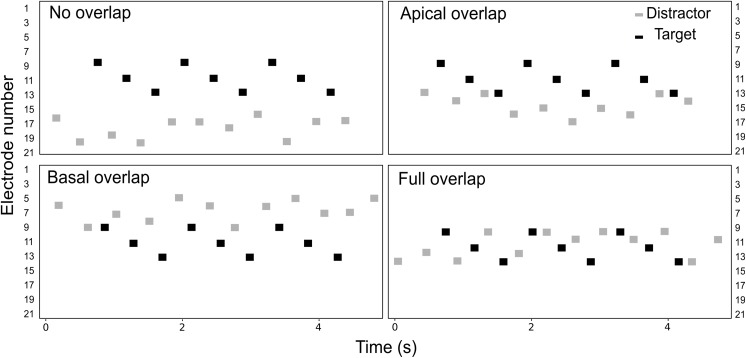
Schematic representation of the electrodogram for each of the four electrode separation conditions. Black and gray markers represent the target and the distractor sounds, respectively. On each trial, the distractor stream started before the target stream and ended after the target stream, with a random number of one to four sounds.

The listeners were asked to perform a detection task, illustrated in **Figure 1**. The target stream consisted on a pattern of sounds, presented on electrodes 9, 11, and 13 (i.e., a *triplet*). On each trial, three triplets were presented consecutively in the presence of interleaved, random distractor sounds which the listeners were asked to ignore. Both the target and the distractor streams extended over a range of five electrodes. The electrode range of the distractor stream was varied across conditions, as illustrated in **Figure 2**. In the *no overlap* condition, the distractor stream was presented through electrodes 16–20, resulting in a separation of three or more electrodes between the streams. In the *apical overlap* condition, the distractor stream was presented through electrodes 13–17, resulting in an overlap with the most apical electrode of the target stream. In the *basal overlap* condition, the distractor stream was presented through electrodes 5–9, such that there was an overlap with the most basal electrode of the target stream. Finally, in the *full overlap* condition, the electrode range of both target and distractor streams was identical (i.e., electrodes 9–13).

Each triplet began with a target sound and ended with a distractor sound. A deviant triplet was randomly introduced in 75% of the trials by reversing the electrode sequence in one of the three triplets (i.e., electrodes 13, 11, and 9). In the remaining 25% of the trials, no deviant was presented, i.e., the three triplets were identical (*no deviant* condition, see **Figure 1**). The four deviant conditions (i.e., deviant triplet 1, 2, 3 or no deviant) were presented in random order. The listeners were asked to detect sequences that contained a deviant and to report its location within the trial in a one-interval, four-alternative forced-choice paradigm.

Behavioral responses during both the training and the data collection were recorded using a custom-made user interface in Python. Four response buttons were used to record the listener’s response 200 ms after each trial. The duration of the inter-trial interval was randomized between 1.5 and 2.5 s. Feedback was provided after each trial.

Each sound consisted of a 50 ms burst of biphasic pulses presented at a given electrode. Each biphasic pulse had a phase width of 25 μs and an inter-phase gap of 8 μs. The pulse rate was fixed at 900 pps. The inter-stimulus interval (ISI) was 440 ms between two consecutive target or distractor sounds, and 220 ms between two consecutive target-distractor sounds (see **Figure 1**). The stimuli were presented in monopolar mode through the Nucleus Implant Communicator research interface (NIC v3, Cochlear Ltd., Sydney) and a research speech processor (L34) provided by Cochlear Ltd.

### Loudness Balancing

Previous studies have suggested that loudness could be an effective cue for the segregation of sounds for CI listeners (e.g., Cooper and Roberts, 2009; Marozeau et al., 2013). To ensure that the listeners did not rely on loudness cues to segregate the sounds, the stimuli of the present study were loudness-balanced. A total of 16 electrodes (from electrode 5 to electrode 20) were used in the present study. Categorical loudness scaling was used to find the most comfortable level (MCL) for six electrodes (i.e., electrodes 5, 8, 11, 14, 17, and 20) using an 11-step attribute scale, as described in Paredes-Gallardo et al. (2018a). The MCL for the remaining electrodes (i.e., electrodes 6, 7, 9, 10, 12, 13, 15, 16, 18, and 19) was obtained by linear interpolation. All electrodes were then loudness matched to a reference electrode (electrode 11) by the listeners, using a simple user interface. The interface allowed the increase and the decrease of the test-sound intensity in steps of 0.15, 0.3, or 0.45 dB.

### Inclusion Criteria

Most CI listeners report a monotonic relation between the place of stimulation and the corresponding pitch percept (e.g., Eddington et al., 1978; Tong et al., 1980; Shannon, 1983; Townshend et al., 1987). However, several previous studies reported instances where the pitch percept did not follow a monotonic function (e.g., Nelson et al., 1995; Collins et al., 1997). In the present study, local pitch reversals could hinder the performance in the detection task. Thus, a pitch ranking experiment was conducted with eight odd-numbered electrodes (between electrodes 5 and 20) using the midpoint comparison procedure (Long et al., 2005; Macherey and Carlyon, 2010). All twelve listeners exhibited monotonic pitch ranks. Furthermore, to ensure that all listeners were able to perform the detection task, a test run with 20 presentations of each of the four different conditions (see **Figure 1**) was performed in the absence of the distractor stream. A minimum average performance of 90% correct was achieved by all listeners.

### Behavioral Experiment

#### Stimuli and Conditions

In the behavioral experiment, the four electrode separation conditions between the target and the distractor streams were tested (i.e., *no overlap*, *apical overlap*, *basal overlap*, and *full overlap* conditions – see **Figure 2**). The target stream was always presented on electrodes 9, 11, and 13, whereas the electrode range of the distractor stream was varied.

In each trial, a random number of one to four distractor sounds was played before and after the target stream (i.e., inducer sounds). Thus, the listeners did not have a priori knowledge about the starting point of the target stream. This was done to encourage the listeners to attend to the full duration of the trial instead of listening for a specific time point. The duration of each trial ranged between 4 and 6.65 s.

#### Procedure

Prior to the behavioral experiment, the listeners underwent 15–20 min of training in the stream segregation task. The training began with the detection task in the absence of the distractor stream. Once the listeners were familiarized with the sequences, the distractor stream was introduced at a soft, but audible, level (*no overlap* condition). The level of the distractor stream was increased in steps of 0.45 dB every third sequence until both streams where played at the listener’s MCL. The training procedure was repeated with the three remaining distractor sets, i.e., *apical*, *basal* and *full overlap*.

In the behavioral experiment, a total of 20 trials were presented for each electrode separation and each of the four deviant conditions. The resulting 320 trials were divided into eight blocks. A block consisted of 10 trials of each of the four deviant conditions for a given electrode separation condition. The order of the blocks was randomized.

#### Data Analysis

The sensitivity measure (d′) was calculated using equation (1) for each of the three deviant triplet locations (*i*), where *z* represents the *z-*transformation, *N_Hi_* and *N_FAi_* the number of hits and false alarms, respectively, and *H* and *FA* the maximum number of hits and false alarms (20 and 60, respectively). The *log-linear rule* was used to avoid undefined extremes when the hit or the false alarm rates take the values of zero or one (Hautus, 1995; Verde et al., 2006).

$$d_{i}^{\prime }=z(\frac{N_{Hi}+0.5}{H+1})-z(\frac{N_{FAi}+0.5}{FA+1})$$

Statistical inference was performed by fitting a mixed-effects linear model to the d’ scores. The experimental variables and their interactions were treated as fixed effects whereas listener-related effects were treated as random effects with random intercepts and slopes. The model was implemented in R (R Core Team, 2015) using the *lme4* library (Bates et al., 2014) and the model selection was carried out with the *lmerTest* library (Kuznetsova et al., 2017) following the backward selection approach based on step-wise deletion of model terms with high *p*-values (Kuznetsova et al., 2015). The *p*-values for the fixed effects were calculated from *F*-tests based on Satterthwaite’s approximation of denominator degrees of freedom and the *p*-values for the random effects were calculated based on likelihood ratio tests (Kuznetsova et al., 2015). The *post hoc* analysis was performed through contrasts of least-square means using the *lsmeans* library (Lenth, 2016). The *p*-values were corrected for multiple comparisons using the Tukey method.

### Recording of Event-Related Potentials

#### Stimuli and Conditions

The *no overlap* condition was chosen for the recording of the ERPs. Thus, the target stream was presented at electrodes 9, 11, and 13 and the distractor stream comprised electrodes 16 to 20. It has been suggested that the first sound of a sequence may draw attention exogenously (e.g., Choi et al., 2014; Dai et al., 2018). In order to ensure that the listeners deployed top-down attention to the target stream, on each trial, a single distractor sound was played before the first triplet. Thus, in the present study, the target stream was always the lagging stream.

A 50 ms burst of pulses on electrode 11, followed by a 750 ms silence was played before each trial. This burst was not relevant for the behavioral task, and the listeners were not given specific listening instructions (whether to attend or ignore it). The burst was included to normalize the N1 amplitude for the remaining sounds across listeners (e.g., Choi et al., 2014). It was hypothesized that the N1 response elicited by this burst would reflect individual differences in the N1 amplitude but would not be affected by attention. However, the N1 responses to this burst were affected by attention, and this effect was variable across listeners. Thus, the responses to this pre-trial burst were not used for the normalization of the individual N1 amplitudes.

#### Procedure

Two attention conditions were tested in the ERP recording session: an *active listening* condition and a *passive listening* condition. During the active listening condition, the scalp electroencephalogram (EEG) was recorded while the listeners performed the behavioral detection task. In the passive listening condition, the EEG signal was recorded in response to the same sounds while the listeners watched a muted movie with captions. Overall, a total of 55 trials were recorded for each attention (active/passive) and deviant condition. For the active listening condition, the 220 trials were divided into two blocks of 110 trials each (∼15 min). For the passive listening condition, the 220 trials were recorded in a single block (∼30 min).

#### EEG Data Acquisition and Analysis

The EEG data were recorded using the Biosemi ActiveTwo^TM^ system at a sampling rate of 8192 Hz. The hardware anti-aliasing filter bandwidth follows a 5th order sinc response, with the -3 dB point located at 1600 Hz. 68 electrodes were used for the recording: 64 electrodes mounted on an elastic headcap according to the international 10–20 electrode configuration, two electrodes at the left and right mastoids, one electrode near the outer canthus of the eye and one electrode below the eye contralateral to the CI. The electrodes directly over the coil were not used in the recording and all electrode wires were directed away from the coil to minimize radio frequency (RF) artifact. The offsets of the recording electrodes were kept below 20 mV in all recordings.

The data were processed using the Fieldtrip toolbox (Oostenveld et al., 2011) and customized Matlab scripts. The continuous EEG data were re-referenced to the average mastoids, highpass-filtered at 1 Hz (FIR with zero-phase lag, 1 Hz transition bandwidth) and lowpass-filtered at 100 Hz (FIR with zero-phase lag, 1 Hz transition bandwidth). The data were downsampled to 256 Hz and epoched from -1.3 to 4.7 s relative to the onset of the first distractor sound. The total duration of the epoch was used for baseline subtraction. Epochs containing unique, non-stereotyped artifacts were manually rejected. Infomax independent component analysis (ICA) was then applied to the remaining epochs. Equivalent current dipole modeling was computed for all independent components (ICs) on each condition. ICs representing eye blinks and saccadic eye movement were manually identified based on their scalp topography, waveform and power-spectrum. Components representing the RF artifact from the implant were automatically identified with a custom implementation of the procedure described in Viola et al. (2012) (see **Supplementary Material** for more details). Artefactual components were removed from all datasets and data were back-projected to the sensor space. After artifact correction, a second baseline subtraction was performed. The time interval between -1.05 and -0.85 s was used for this second baseline subtraction. Epochs were lowpass-filtered at 20 Hz (FIR, zero-phase lag, 1 Hz transition bandwidth and 1 s zero-padding both before and after the epoch).

Only the correctly answered trials were processed. Since there were not enough correct trials to analyze the data for each of the four deviant conditions, the epochs were grouped in *early* and *late* deviant conditions. The *early* deviant group contained the epochs where either the first or the second triplets were a deviant. The *late* deviant group contained the epochs where the deviant was in the third triplet or absent (i.e., the epochs where the listeners had to sustain selective attention throughout the full duration of the sequence). A minimum of 64 correct trials was available for each listener, deviant condition (*early* vs. *late*) and attention condition (active vs. passive). Thus, the first 64 correctly answered trials for each listener, deviant condition and attention condition were analyzed. The deviant count was balanced within each deviant condition for all listeners except for CI-6 (i.e., 32 trials per deviant triplet). For CI-6, only 25 correct trials of the first deviant triplet were available, which were pooled with 39 trials of the second deviant triplet on the *early* deviant condition.

For each condition, listener and electrode, the amplitude of the N1 ERP component was calculated as the local minimum in the time window from 70 to 170 ms after each sound onset (i.e., both for the target and the distractor sounds). For each listener, the across-electrode N1 amplitude was calculated by averaging the amplitudes from nine front-central electrodes (Fz, AFz, FCz, F1, F2, FC1, FC2, AF3, and AF4). This average measure will be referred to as N1 amplitude. The attentional modulation of the ERPs was quantified for each listener as the difference in N1 amplitude between the active and passive listening conditions for each sound. Thus, a negative value indicates a larger N1 response in the active than in the passive listening condition. These values were averaged across all target sounds to obtain a single estimate per listener. The single value was used to compute the Kendall rank correlations between the behavioral performance and the attentional modulation of the ERPs.

A mixed-effects linear model was used for the statistical analysis. N1 amplitude differences between the active and the passive listening conditions were modeled following the approach described in the section “Data Analysis.”

## Results

### Behavioral Experiment

**FIGURE 3:**
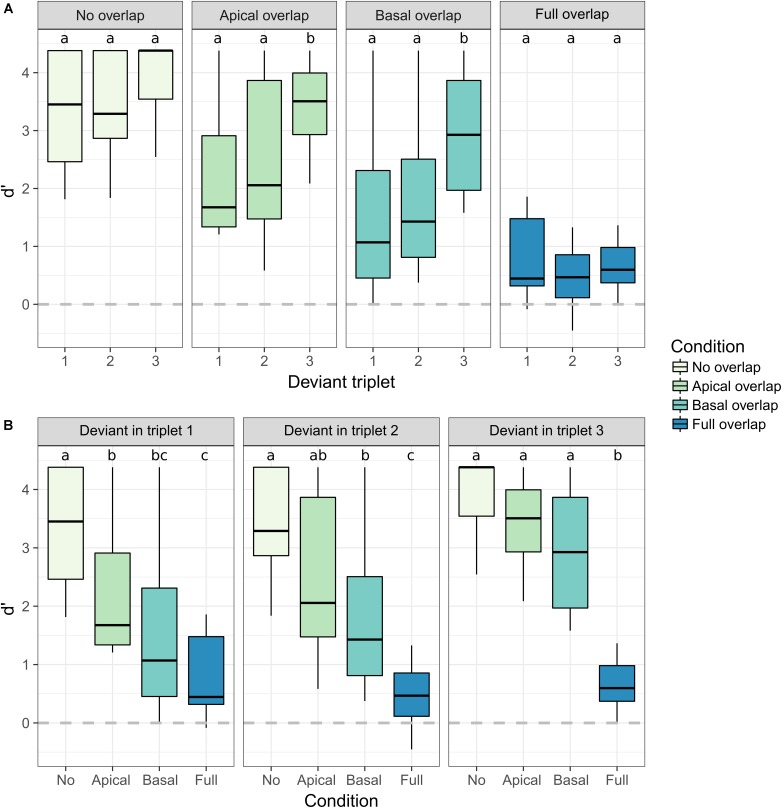
Boxplot of the sensitivity scores (d′) to the deviant triplet for each electrode separation and deviant triplet location. The color of the boxes represents the electrode separation condition. **(A)** Effect of the deviant triplet for each electrode separation condition. **(B)** Effect of the electrode separation for each deviant triplet location. Results from the statistical contrasts are indicated with lowercase letters. Conditions sharing one or more letters are not significantly different (significance level α = 0.05).

The results from the behavioral experiment are shown in **Figure 3**. The d′ scores are shown for each combination of electrode separation (A) and deviant triplet location (B). Different electrode separation conditions are shown with different colors. Statistically significant differences between conditions are illustrated with letters. Conditions sharing one or more letters are not significantly different. Detailed statistics from the *post hoc* analysis are provided in the **Supplementary Material**.

Overall, the d′ scores increased the later the deviant triplet occurred [*F*(2,11.75) = 16.423, *p* < 0.001] and decreased with increasing electrode overlap between the streams [*F*(3,11.39) = 73.484, *p* < 0.001]. Moreover, a significant interaction was found between the deviant triplet location and the electrode overlap between the streams [*F*(6,88.00) = 5.811, *p* < 0.001], indicating that the effect of the deviant triplet location was not the same for all electrode separation conditions.

The location of deviant triplet did not affect the d′ scores for the *no overlap* condition (**Figure 3A**). However, the d′ scores were at ceiling for this condition, preventing any effect of the deviant triplet location to be observed. Similarly, no effect of the deviant triplet location was observed for the *full overlap* condition, where the d′ scores were close to zero. The largest effect of the deviant triplet location was observed for the *apical* and *basal overlap* conditions. In these conditions, significantly larger d′ scores were achieved when the deviant triplet happened at the end of the sequence.

When the deviant occurred in the first triplet, the d′ scores for the *no overlap* condition were significantly larger than the ones achieved for any of the other conditions (**Figure 3B**). The difference between the *no overlap* condition and the *apical* and *basal overlap* conditions was reduced when the deviant occurred in the second triplet. No significant difference was observed between these three conditions when the deviant occurred in the third triplet. The d′ scores were generally lower for the *basal overlap* than for the *apical overlap* condition. However, no significant difference was observed between these two conditions for any of the deviant triplet locations.

### Event-Related Potentials

**FIGURE 4:**
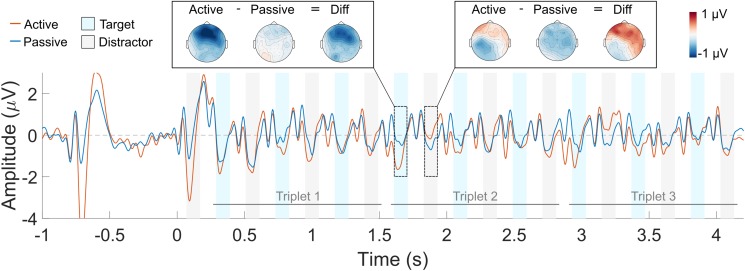
Averaged ERP waveform across the four deviant triplet conditions. The active listening condition is shown in red and the passive listening condition in blue. The blue and gray shaded areas indicate the N1 ERP component time window for the target and the distractor sounds, respectively. Each trace represents the average across nine front-central electrodes (Fz, AFz, FCz, F1, F2, FC1, FC2, AF3, AF4). The scalp topography of the response to a target and a distractor sound is shown for each of the listening conditions and their difference.

**FIGURE 5:**
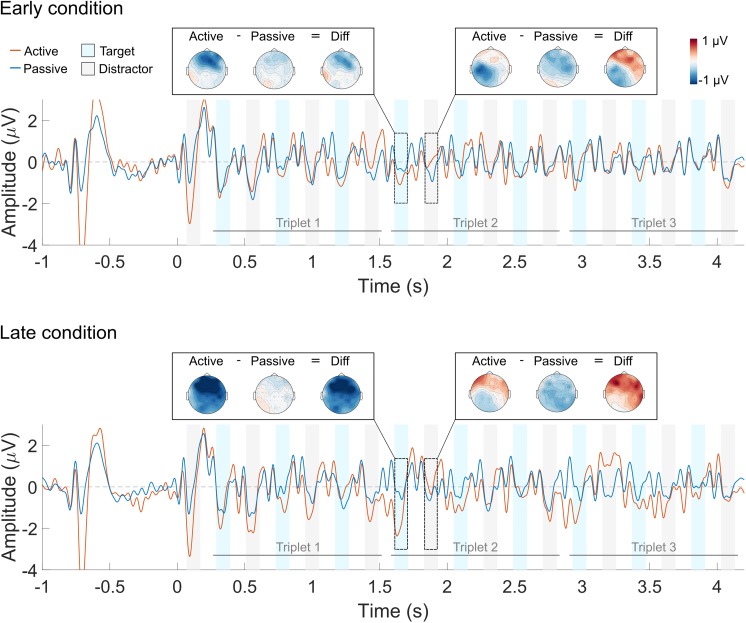
Averaged ERP waveform for the *early* **(top)** and *late* **(bottom)** deviant conditions. The active listening condition is shown in red and the passive listening condition in blue. The blue and gray shaded areas indicate the N1 ERP component time window for the target and the distractor sounds, respectively. Each trace represents the average across nine front-central electrodes (Fz, AFz, FCz, F1, F2, FC1, FC2, AF3, AF4). The scalp topography of the response to a target and a distractor sound is shown for each of the listening conditions and their difference.

The grand average waveform across all listeners is shown in **Figure 4** (averaged across all deviant conditions) and in **Figure 5** (in separate panels for the *early* and *late* deviant conditions). Red and blue solid lines represent the active and the passive listening conditions, respectively. Blue and gray shaded areas indicate the N1 response time window for the target and the distractor sounds, respectively. Sharp oscillations before the N1 time window are likely to represent the residual CI artifact, and should not be mistaken for a P1 response. The scalp distribution of the response to one target and one distractor sound is also shown for each listening condition and their difference. The scalp distributions were obtained by averaging the response over the N1 time window for each of the 64 electrodes. Blue and red colors represent negative and positive values, respectively.

N1 responses to the first triplet are, qualitatively, similar for the active and the passive listening condition (**Figure 4**). This was the case for both the target and the distractor sounds. Conversely, the N1 attentional modulation seem, qualitatively, different for the target and the distractor sounds in the second and third triplets: selective attention enhanced the N1 responses to the target sounds (i.e., the N1 amplitude is more negative in the active vs. the passive listening condition) and suppressed the N1 responses to the distractor sounds (i.e., the N1 amplitude is more negative in the passive vs. the active listening condition). However, this was only observed for the first two target sounds and the first distractor sound of the second and third triplets. Similar patterns can be seen in **Figure 5**, both for the *early* and for the *late* deviant conditions. Nevertheless, the effect of attention is largest for the *late* deviant condition. This is apparent when comparing the topography of the N1 responses to a target and a distractor sound in **Figures 4**, **5**.

The individual N1 attentional modulation for each deviant condition (*early* vs. *late* deviant), sound type (target vs. distractor), triplet number and sound number (first, second, or third sound of a triplet) was modeled using a mixed-effects statistical model. The first sound of the sequence was not part of any of the triplets and therefore, was excluded from the analysis. The model revealed a significant main effect of the sound type [*F*(1,356) = 20.051, *p* < 0.001]. Moreover, a significant interaction was found between the triplet number, the deviant condition and the sound type [*F*(2,356) = 4.557, *p* = 0.011] and between the sound number, the deviant condition and the sound type [*F*(2,356) = 3.111, *p* = 0.046]. No significant interaction was found between the triplet number, the sound number and the deviant condition [*F*(4,344) = 0.464, *p* = 0.763] or between the triplet number, the sound number, the sound type and the deviant condition [*F*(4,340) = 1.049, *p* = 0.382].

A *post hoc* analysis revealed that the N1 responses to the target sounds were, on average, enhanced by 0.623 μV in the active vs. the passive listening condition [*t*(14.9) = 4.336, *p* = 0.001]. Conversely, the difference in the N1 responses elicited by the distractor sounds was not statistically significant [estimate = 0.075 μV, *t*(14.9) = 0.524, *p* = 1]. This was also the case when including the responses to the first sound of the sequence (i.e., a distractor sound) in the analysis.

**FIGURE 6:**
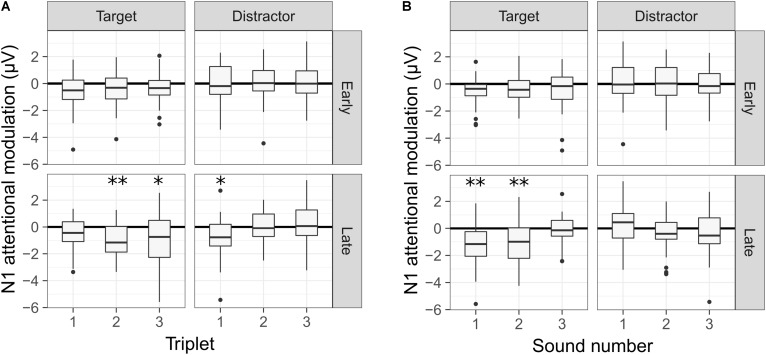
N1 attentional modulation of the target and distractor sounds for each deviant condition. A negative value represents an enhanced N1 response in the active condition. **(A)** Averaged N1 attentional modulation across the three sounds of each triplet. **(B)** Averaged N1 attentional modulation across the three triplets for each sound. A statistically significant difference from zero is indicated by one asterisk if 0.05 > *p* > 0.01, two asterisks if 0.01 > *p* > 0.001 and three asterisks if *p* < 0.001. The *p*-values were corrected for multiple comparisons using the Bonferroni correction.

The significant interactions from the statistical model are illustrated in **Figure 6**, where the N1 attentional modulation is shown for each sound type and deviant condition. In **Figure 6A**, the N1 attentional modulation is averaged across the three sounds of each triplet, illustrating the interaction between the triplet number, the deviant condition and the sound type. For the *early* deviant condition, no significant modulation of the N1 responses was observed for either the target or the distractor sounds in any of the three triplets. For the *late* deviant condition, a significant N1 enhancement was observed for the first triplet of the distractor stream [*t*(26.97) = 2.719, *p* = 0.034] and for the second [*t*(26.97) = 3.315, *p* = 0.008] and third [*t*(26.97) = 2.672, *p* = 0.038] triplets of the target stream. In **Figure 6B**, the N1 attentional modulation is averaged across the three triplets for each sound, illustrating the interaction between the sound number, the deviant condition and the sound type. As in **Figure 6A**, no significant modulation of the N1 responses to any of the sounds was observed for the *early* deviant condition. However, a significant N1 enhancement was observed for the first [*t*(26.97) = 3.763, *p* = 0.003] and second [*t*(26.97) = 3.451, *p* = 0.006] target sounds for the *late* deviant condition.

**FIGURE 7:**
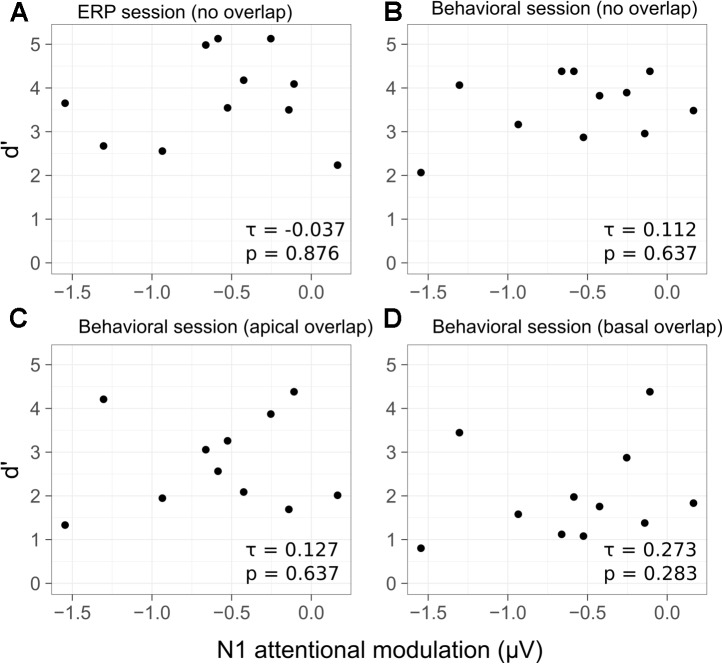
Scatterplots of the d′ scores as a function of the average N1 attentional modulation for each listener and condition. Behavioral d′ scores from the ERP session are shown in **(A)** (*no overlap* condition). The d′ scores from the behavioral session are shown in **(B)** for the *no overlap* condition, in **(C)** for the *apical overlap* condition and in **(D)** for the *basal overlap* condition. Kendall rank correlation coefficients and *p*-values are shown in the bottom-right corner of each panel.

The relation between the individual d′ scores and the N1 attentional modulation of the target sounds is shown in **Figure 7**. No significant correlation was found between the d′ scores achieved in the active listening condition and the N1 attentional modulation (**Figure 7A**) [τ = -0.037, *p* = 0.876]. Kendall rank correlation scores were also computed for the N1 attentional modulation and the d′ scores from the behavioral session. No significant correlation was found for the *no overlap* condition (**Figure 7B**) [τ = 0.112, *p* = 0.637], for the *apical overlap* condition (**Figure 7C**) [τ = 0.127, *p* = 0.648] or for the *basal overlap* condition (**Figure 7D**) [τ = 0.273, *p* = 0.283].

## Discussion

### The Effect of Electrode Separation on Stream Segregation

Performance in the detection task was assumed to improve when the target and the distractor streams were perceptually segregated. Overall, the d′ scores obtained by the listeners increased with increasing electrode separation between the streams. The d′ scores obtained by the listeners were near chance-level for the *full overlap* condition, indicating that the listeners could not segregate the streams in the absence of place cues. Conversely, performance was at ceiling for the *no overlap* condition, suggesting that the listeners were able to use place cues to segregate the streams. These findings are consistent with previous work suggesting that electrode separation facilitates stream segregation for CI listeners (Chatterjee et al., 2006; Hong and Turner, 2006; Böckmann-Barthel et al., 2014; Tejani et al., 2017; Paredes-Gallardo et al., 2018a).

Previous studies generally assessed stream segregation using sequences composed of two repeating and alternating sounds. In such paradigms, the listeners need to segregate the target sound from the distractor sound. In contrast, in the present study, each stream was composed of multiple and different sounds, increasing the complexity of the task: the listeners had to integrate different sounds to form a representation of the target and the distractor streams and to maintain these representations segregated over time. Despite the differences between the paradigms, the results of the present study were consistent with those from Paredes-Gallardo et al. (2018a), which suggested that most CI listeners can segregate streams separated by three electrodes. In the present study, all listeners could segregate the streams in the *no overlap* condition, where the minimum electrode separation between the streams was three electrodes.

The d′ scores obtained by the listeners increased for deviants happening late in the sequence. This suggests that a two stream percept built up over time (for a review, see Moore and Gockel, 2002, 2012). These results are consistent with earlier reports suggesting that CI listeners may experience a build-up when attention is directed toward segregation, i.e., voluntary stream segregation (Paredes-Gallardo et al., 2018a,b). Moreover, the effect of the deviant triplet location was found to be dependent on the electrode separation between the streams. The largest effect of the deviant triplet location (i.e., build-up) was observed for the *apical overlap* and the *basal overlap* conditions, where the performance was not at ceiling or at chance level. This is consistent with previous reports from studies with NH listeners (e.g., Bregman, 1978; Anstis and Saida, 1985) and CI listeners (Böckmann-Barthel et al., 2014), that reported a build-up for intermediate frequency differences between the sounds. However, Böckmann-Barthel et al. (2014) did not provide specific listening instructions to the participants, who directly reported their perception. It has been suggested that the results from such paradigms may reflect pitch or electrode discrimination instead of stream segregation (Chatterjee et al., 2006; Cooper and Roberts, 2007). This uncertainty was avoided in the present study by using a detection task which was facilitated by the segregation of the sounds (e.g., Dowling, 1973; Cusack and Roberts, 2000; Cooper and Roberts, 2009).

In the behavioral session, a random number of distractor sounds was played, in each trial, before and after the target stream (i.e., inducer sounds). It has been suggested that inducer sounds can trigger the build-up and therefore, may facilitate stream segregation (e.g., Rogers and Bregman, 1993; Roberts et al., 2008). To evaluate whether the presence of a random number of inducer sounds facilitated stream segregation, the d′ scores from the behavioral *full overlap* condition were compared with those from the ERP recording session, where no inducer sounds were presented. The results from a mixed-effects linear model showed no significant effect of the inducer sounds [*F*(1,11) = 2.701, *p* = 0.129], indicating that these did not affect the d′ scores.

Despite the similarity of the paradigms, the findings from the present study appear to be inconsistent with those reported by Cooper and Roberts (2009). In their study, the electrode separation between the streams did not affect the performance in the melody discrimination task. Moreover, most of their listeners performed near-chance level unless the distractor sounds were attenuated by at least 50% of the listener’s dynamic range. In contrast, in the present study, all listeners were able to use electrode separation to segregate the streams when the target and the distractor stream were presented at the same loudness. Cooper and Roberts employed sequences with a fixed duration of 2.2 s whereas in the present study, the sequences had a duration which ranged between 4 and 6.65 s. Therefore, one might argue that in the study from Cooper and Roberts, the listeners did not have enough time to build up a segregated percept, as suggested by Paredes-Gallardo et al. (2018a). If this was the case, in the present study, near-chance performance would be expected for the first deviant triplet, which happened between 1.3 and 2.6 s. Instead, the results from the behavioral experiment indicate that the effect of the electrode separation on the d′ scores was largest for the first deviant triplet: the d′ scores ranged from near-chance in the *full overlap* condition to near-ceiling in the *no overlap* condition.

In the study of Cooper and Roberts (2009) pitch direction judgments were required to identify the target melody. In contrast, in the present study, the listeners were not required to identify the melodic contour of the target stream, and could, instead, perform the task by detecting a change (i.e., deviant) in the target stream. Kong et al. (2004), suggested that as many as 32 independent frequency bands are needed to recognize familiar melodies. The spectral resolution of CIs is limited both by the number of electrodes (typically 22 or less) and the interaction of the electrical current across electrodes. Thus, current CIs may not provide enough spectral resolution to support melody recognition (e.g., Kong et al., 2004; Mehta and Oxenham, 2017). Therefore, the poor performance observed by Cooper and Roberts (2009) could reflect inherent limitations of CI listeners to recognize familiar melodies and may not be related to poor stream segregation.

### The Effect of Selective Attention on the ERPs

The N1 responses to the target and the distractor sounds were recorded while the listeners performed the behavioral task (i.e., active listening) and when the listeners watched a muted movie (i.e., passive listening). Even though the same physical stimuli were presented in both conditions, at the group level and when averaged across all deviant conditions, the N1 amplitudes were different in the active and in the passive listening conditions. Thus, selective auditory attention modulated the ERPs. Consistent with previous work in NH and HI listeners, the N1 responses to the target sounds were enhanced when the listeners performed the behavioral task vs. when they passively listened to the sounds (e.g., Choi et al., 2014; Dai and Shinn-Cunningham, 2016; Dai et al., 2018). However, attention did not significantly modulate the responses to the distractor sounds. This is consistent with previous studies suggesting that hearing impairment may affect the ability to suppress irrelevant sounds (Petersen et al., 2017; Dai et al., 2018).

The effect of selective attention on the N1 responses was largest in the *late* deviant condition, where the listeners had to sustain selective attention throughout the full duration of the sequence. In this condition, the N1 responses to the target sounds were enhanced during the second and third triplets, but not during the first one. Conversely, the N1 responses to the distractor sounds were enhanced for the first triplet, but not for the second and third ones (**Figure 6A**). Thus, whereas the attentional modulation of the N1 responses to the target sounds became more robust over time, the attentional modulation of the N1 responses to the distractor sounds diminished over time. These results suggest that CI listeners become more effective at selectively listening to the target stream over time, in agreement with previous reports with NH listeners (e.g., Shinn-Cunningham and Best, 2008; Choi et al., 2014; Dai et al., 2018). Moreover, only the N1 responses to the first two sounds of the target stream were enhanced in the active listening condition (**Figure 6B**). Given the design of the stimuli, only the first two sounds of each triplet were necessary to detect the deviant. Thus, these results suggest that the listeners may have generally relied on the first two target sounds of each triplet to perform the task.

It has been suggested that the use of a cue to direct the listeners’ attention toward a specific attribute of the sound (e.g., location or pitch) leads to anticipatory modulation of the cortical responses (Hill and Miller, 2010; Lee et al., 2013). As a result, the responses evoked by a sound will be larger when its attributes match those of the cue than when there is a mismatch. However, other studies have suggested that the inherent salience of sudden onsets may override this anticipatory modulation, leading to similar ERP responses in the active and the passive listening conditions (e.g., Dai et al., 2018). In the present study, the target stream was temporally predictable (i.e., it was always the lagging stream) and the listeners were familiar with both the target and the distractor stream. Thus, one would expect either a suppression effect on the N1 response to the first sound of the sequences (i.e., a distractor sound) or a lack of any attentional modulation. Instead, a larger N1 response to the first sound was observed for the active than for the passive listening condition (**Figures 4**, **5**). These results suggest that CI listeners might not be able to ignore the leading stream and therefore deploy top-down attention to the first distractor sound. As a consequence, they need to switch their attention from the distractor to the target stream during the trial. Consequently, the N1 attentional modulation of the distractor stream is significantly different from zero during the first triplet whereas the N1 attentional modulation of the target stream is significantly different than zero during the second and third triplets.

### The Relation Between Behavioral Performance and the N1 Attentional Modulation

It has been suggested that selective attention can operate at early stages of sensory analysis, even before the features of the stimulus are bound together, or *conjoined* (e.g., Woods et al., 1994, 1998). In addition, it has been suggested that when attention is focused on a particular stimulus feature, all coherent perceptual features may be bound together forming a stream (e.g., Shamma et al., 2011, 2013). Other studies have suggested that selective attention may operate at the level of auditory objects, even if attention is initially focused on a particular feature (e.g., Alain and Arnott, 2000; Shinn-Cunningham, 2008; Bressler et al., 2014). The presence of a salient perceptual feature in the target stream may, therefore, facilitate the process of object formation. A clear object representation might, in turn, make the process of selective attention more effective. Correspondingly, several studies have found a significant correlation between the attentional modulation of cortical responses and the behavioral performance in a selective auditory attention task (e.g., Choi et al., 2014; Dai and Shinn-Cunningham, 2016; Dai et al., 2018). In the present study, no significant correlation was found between the individual performance in the deviant detection task and the amount of N1 attentional modulation. This was the case for all electrode separation conditions (**Figure 7**). The d′ scores in the *no overlap* condition (**Figures 7A,B**) were, overall, at ceiling. This limited the individual variability of the d′ scores, presumably contributing to the non-significant correlation between the behavioral performance and the N1 attentional modulation. In order to avoid ceiling effects in the behavioral performance while ensuring that enough correct trials are available to estimate the N1 response amplitude, Choi et al. (2014) correlated the behavioral performance in a challenging task with the N1 attentional modulation recorded under a less challenging condition. Choi et al. (2014) found a significant correlation between the behavioral performance and the amount of N1 attentional modulation. In contrast, in the present study, no significant correlation was found between the behavioral performance in the apical and basal overlap conditions, where no ceiling effects were present, and the amount of N1 attentional modulation (**Figures 7C,D**).

The lack of a significant correlation between the d′ scores and the N1 attentional modulation does not necessarily imply the independence of these two measures. Instead, it might reflect the limitations imposed by the conditions and paradigm chosen for the ERP recordings. The d′ scores from the ERP recordings (*no overlap* condition) were at ceiling for most listeners, suggesting that the task was not demanding. Thus, it is possible that some listeners could have achieved high d′ scores in the task without selectively attending to the target stream (e.g., they could have switched their attention between the streams). If this was the case, the individual differences in the N1 attentional modulation would not reflect differences in the ability to perceptually group the sounds. Moreover, the ERP recordings took place in the last of the three sessions. As a result, all listeners were familiar with the target stream at the time of the ERP recordings. During the passive listening condition, the listeners watched a muted movie and after the recording session, the listeners were asked informal questions about the movies. However, the individual level of engagement in the movie was not quantified. Thus, some listeners might have been engaged in the movie whereas other might have attended to the sound sequences. As a result, the N1 attentional modulation estimates from the present study might not be precise at an individual level.

## Conclusion

The present study combined a behavioral deviant detection task with ERP recordings to investigate the role of electrode separation in voluntary stream segregation for CI listeners. The results suggested that CI listeners can voluntarily segregate streams composed of multiple and different sounds when only electrode separation cues are provided. Moreover, a two-stream percept was found to build up over time. The results from the ERP recordings showed that auditory selective attention modulates the cortical responses in CI listeners. Specifically, selective attention enhanced the responses to the target sounds whereas responses to the distractor sounds remained unchanged. However, no correlation was found between the behavioral performance in the detection task and the attentional modulation of the ERPs.

Overall, the results from the present study suggest that CI listeners can perceptually group sequentially presented sounds into streams on the basis of electrode separation. Moreover, the effects of selective attention could be measured in CI listeners using EEG recordings (at the group level). This suggests that CI listeners, like NH listeners, are able to selectively attend to a target stream as long as its distinctive feature is sufficiently salient. The results from the present study also suggest that CI listeners might experience limitations in their ability to ignore a competing stream and to initially select the stream of interest, which might contribute to their poor performance in complex listening scenarios.

## Data Availability

The data generated for the present study is publicly available at https://doi.org/10.5281/zenodo.1211574. The code used for the CI artifact correction is publicly available at https://doi.org/10.5281/zenodo.1303275.

## Author Contributions

AP-G, HI-B, SM, TD, and JM designed the research. AP-G performed the research. AP-G and HI-B analyzed the data. AP-G, HI-B, SM, TD, and JM wrote the paper.

## Conflict of Interest Statement

The authors declare that the research was conducted in the absence of any commercial or financial relationships that could be construed as a potential conflict of interest.
