# Supplementary material for: Auditory Stream Segregation and Selective Attention for Cochlear Implant Listeners: Evidence From Behavioral Measures and Event-Related Potentials
Source: Front Neurosci. 2018 Aug 21;12:581. doi: 10.3389/fnins.2018.00581 (PMC6110823; doi:10.3389/fnins.2018.00581)
Supplement: Supplementary file 1 [file Data_Sheet_1.pdf]

Supplementary Material

Electrode separation as a cue for auditory stream segregation in cochlear implant listeners: Evidence from behavioral measures and event-related potentials

Andreu Paredes-Gallardo*, Hamish Innes-Brown, Sara Miay Kim Madsen, Torsten Dau, Jeremy Marozeau

*** Correspondence:** Andreu Paredes-Gallardo: apaga@elektro.dtu.dk

1 Cochlear implant artifact attenuation

The cochlear implant (CI) artifact was attenuated using a custom implementation of the procedure proposed by Viola et al. (2012). This approach makes use of independent component analysis (ICA) to decompose the electroencephalographic (EEG) signal into statistically maximally independent components. Instead of manually selecting the independent components (ICs), Viola et al. (2012) proposed three criteria to distinguish between ICs representing the neural activity, such as the N1 response, and the CI artifact: 1] The topography of ICs representing neural activity is well modeled with a dipole. Thus, the residual variance (RV) between the projection of the equivalent dipole model and the actual topography of the IC is generally low. Conversely, ICs representing the CI artifact exhibit less dipolar topographies and thus, a larger RV. 2] The largest activity of the N1 response is generally observed about 100 ms after the onset of the stimuli. Conversely, the largest activity of the CI artifact happens at the onset and offset of the stimulation. 3] ICs representing the artifact of a particular listener generally present similar topographies.

Based on these three criteria, Viola et al. (2012) proposed an algorithm to identify the ICs which represent the CI artifact. The algorithm consists of three steps. First, ICs with RV above a threshold are selected. Second, the first derivative of the selected ICs is calculated. The ratio between the root mean square (RMS) amplitude in the stimulus onset/offset time window and in the time window where the response of interest is expected (e.g. N1) is computed. The IC with the largest ratio is chosen as the topographical template for the CI artifact. The topography of the template is then correlated with the remaining ICs. In the third step, ICs either exceeding a ratio-threshold or a correlation-threshold are selected as CI components.

The algorithm proposed by Viola et al. (2012) was designed to attenuate the CI artifact from EEG responses to single sounds which could overlap with the time window of the neural response. In the present study each trial consisted on 19 single electrode stimuli, each with a duration of 50 ms. For this reason, the artifact onset and offset time windows were replaced by a single time window from -10 to +60 ms (relative to the sound onset). As a result, a total of 19 RMS ratios were calculated in the second step. These values were averaged to obtain a single measure of the ratio between CI artifact and response for each IC. The RV-threshold was set to 10%, the ratio-threshold to 2.7 and the correlation –threshold to 0.85. The code used for the CI artifact correction is publicly available at https://doi.org/10.5281/zenodo.1303275.

The algorithm was applied for each listener and listening condition (i.e. active and passive listening) independently. Thus, the artifact attenuation process could have introduced variations in the EEG waveforms that could be confounded with an attentional effect. To ensure that this was not the case, the ICs representing the artifact were back-projected to the sensor space (i.e. artefactual data). The clean data (i.e. data from which ICs representing the artefact had been removed) and the artefactual data were then processed in the same way.

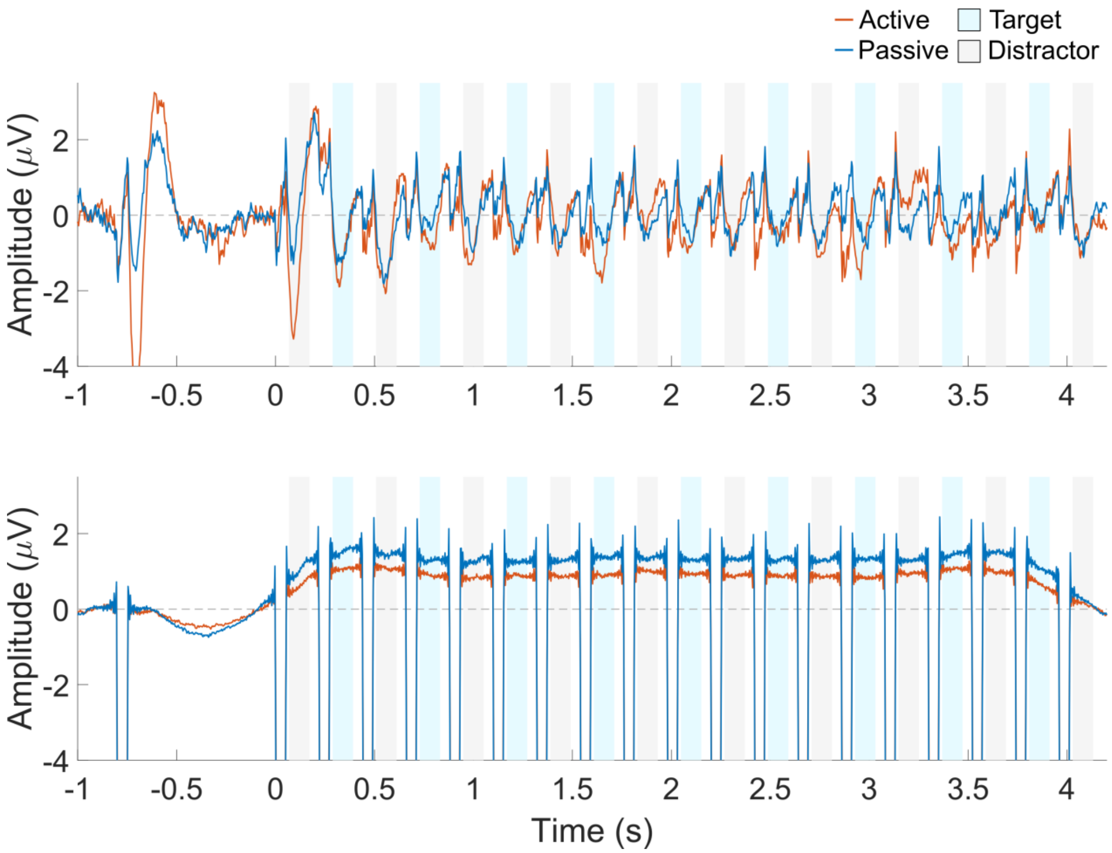

Supplementary Figure 1 Grand average waveform for the clean data (top) and the artefactual data (bottom). The active listening condition is shown in red and the passive listening condition in blue. The blue and gray shaded areas indicate the N1 ERP component time window for the target and the distractor sounds, respectively. Each trace represents the average across nine front-central electrodes (Fz, AFz, FCz, F1, F2, FC1, FC2, AF3, AF4).

The grand average waveform for the clean and the artefactual data is shown in Supplementary Figure 1. Red and blue solid lines represent the active and the passive listening conditions, respectively. Blue and gray shaded areas indicate the N1 response time window for the target and the distractor sounds, respectively. For the clean data (top panel), most of the activity is observed in the N1 response time windows. Sharp peaks can still be seen in the clean data, just before the N1 response time windows. This indicates that the CI artifact was not totally removed by the algorithm. For the artefactual data (bottom panel), little activity is observed in the N1 time windows. Instead, large square pulses are observed just before the N1 response time window, representing the CI artifact. These results imply that the artifact attenuation process did not introduce significant variations in the EEG waveform.

2 Detailed results from the post hoc analysis of the behavioral experiment

Table 1 Results from the pairwise comparison between the d' scores achieved for each deviant triplet and electrode separation condition. Independent comparisons were performed for each electrode separation condition. Reported p-values have been corrected for multiple comparisons using the Tukey method for a family of 12 estimates.

Electrode separation	Deviant triplet	Estimate	df	t ratio	p-value
No overlap	1 – 2	-0.005	41.86	-0.021	1
	1 – 3	-0.614	49.55	-2.677	0.268
	2 – 3	-0.609	37.20	-2.419	0.419
Apical overlap	1 – 2	-0.272	41.86	-1.126	0.992
	1 – 3	-1.210	49.55	-5.276	<0.001
	2 – 3	-0.937	37.20	-3.725	0.027
Basal overlap	1 – 2	-0.219	41.86	-0.907	0.999
	1 – 3	-1.457	49.55	-6.354	<0.001
	2 – 3	-1.237	37.20	-4.917	0.001
Full overlap	1 – 2	0.235	41.86	0.974	0.998
	1 – 3	0.071	49.55	0.309	1
	2 – 3	-0.165	37.20	-0.654	1

Table 2 Results from the pairwise comparison between the d' scores achieved for each deviant triplet and electrode separation condition. Independent comparisons were performed for each deviant triplet condition. *No* stands for *no overlap*, *Apcl* for *apical overlap*, *Bsal* for *basal overlap* and *Full* for *full overlap*. Reported p-values have been corrected for multiple comparisons using the Tukey method for a family of 12 estimates.

Deviant triplet	Electrode separation	Estimate	df	t ratio	p-value
1	No – Apcl	1.120	24.16	3.908	0.026
	No – Bsl	1.843	21.95	6.075	< 0.001
	No – Full	2.612	27.52	9.746	<0.001
	Apcl – Bsl	0.723	26.33	2.641	0.308
	Apcl – Full	1.493	25.03	5.311	<0.001
	Bsl – Full	0.769	20.67	2.436	0.426
2	No – Apcl	0.852	24.16	2.975	0.177
	No – Bsl	1.628	21.95	5.369	0.001
	No – Full	2.853	27.52	10.643	<0.001
	Apcl – Bsl	0.776	26.33	2.834	0.222
	Apcl – Full	2.000	25.03	7.117	<0.001
	Bsl – Full	1.224	20.67	3.876	0.032
3	No – Apcl	0.524	24.16	1.827	0.789
	No – Bsl	1.000	21.95	3.296	0.100
	No – Full	3.296	27.52	12.299	<0.001
	Apcl – Bsl	0.476	26.33	1.739	0.835
	Apcl – Full	2.773	25.03	9.866	<0.001
	Bsl – Full	2.297	20.67	7.272	<0.001

3 References

Viola, F. C., De Vos, M., Hine, J., Sandmann, P., Bleeck, S., Eyles, J., & Debener, S. (2012). Semi-automatic attenuation of cochlear implant artifacts for the evaluation of late auditory evoked potentials. *Hearing Research*, *284*(1–2), 6–15. http://doi.org/10.1016/j.heares.2011.12.010
